# A Multi-Core Object Detection Coprocessor for Multi-Scale/Type Classification Applicable to IoT Devices

**DOI:** 10.3390/s20216239

**Published:** 2020-10-31

**Authors:** Peng Xu, Zhihua Xiao, Xianglong Wang, Lei Chen, Chao Wang, Fengwei An

**Affiliations:** 1 School of Microelectronics, Southern University of Science and Technology, Shenzhen 518055, China; 11710124@mail.sustech.edu.cn (P.X.); 11713016@mail.sustech.edu.cn (Z.X.); 12031015@mail.sustech.edu.cn (X.W.); 2 Pengcheng Laboratory, Shenzhen 518055, China; chenl03@pcl.ac.cn; 3 Department of Integrated Circuit Engineering, Huazhong University of Science and Technology, Wuhan 430074, China; chao_wang_me@hust.edu.cn; 4 Wuhan National Laboratory for Optoelectronics, Wuhan 430074, China; 5 Engineering Research Center of Integrated Circuits for Next-Generation Communications, Ministry of Education, Southern University of Science and Technology, Shenzhen 518055, China

**Keywords:** power efficiency, object-detection coprocessor, histogram of oriented gradient, support vector machine, block-level once sliding detection window, multi-shape detection-window

## Abstract

Power efficiency is becoming a critical aspect of IoT devices. In this paper, we present a compact object-detection coprocessor with multiple cores for multi-scale/type classification. This coprocessor is capable to process scalable block size for multi-shape detection-window and can be compatible with the frame-image sizes up to 2048 × 2048 for multi-scale classification. A memory-reuse strategy that requires only one dual-port SRAM for storing the feature-vector of one-row blocks is developed to save memory usage. Eventually, a prototype platform is implemented on the Intel DE4 development board with the Stratix IV device. The power consumption of each core in FPGA is only 80.98 mW.

## 1. Introduction

Real-time processing ability is required by multiple tasks such as auto-drive, Internet of Things (IoT) systems, security systems, and so on. Even though the CPU (Central Processing Unit) and GPU (Graphics Processing Unit)-based solutions are flexible and can be easily used on multiple devices for different tasks, its low processing speed and high-power consumption make it inefficient for edge computation devices. Typically, the edge devices using low-power processors find it hard to handle complex tasks or have to compromise on precision and speed. Meanwhile, hardware-friendly VLSI (Very Large-Scale Integration) becomes attractive for satisfying the speed and power need of edge devices. Even the VLSI has an undesirable design complexity and flexibility loss. The high performance and low power consumption of VLSI implementation are suitable to the edge device which needs to handle real-time tasks.

### 1.1. Related Work

Vehicle detection is extremely important for driverless cars or advanced driver assistance systems (ADAS). With the development of Convolutional Neuron Networks (CNN), many researchers are working on using CNN for object detection (e.g., YOLO [1], RFCN [2], VGG [3]), but only a small subset of papers discuss the running time in any detail. For example, the design in [4,5] have a considerable accuracy in detection tasks but incur high computation costs to execute their CNN models. Furthermore, these papers only claim the frame rate they achieve but do not give a full picture of the speed-power-accuracy trade-off [6]. Though the speed performance of CNN on the GPU platform has improved a lot, the high memory usage and computation complexity still make it inapplicable for time-critical and low-power-consumption devices.

Nakahara et al. [7] proposed a YOLO-based object detection architecture on FPGA. To make the complex CNN network more hardware friendly, they chose to use a light weighted YOLOv2 and further simplified it to binary weight. According to their paper, for an input image size of 224 × 224, the detection frame rate is 40 fps. Though their FPGA (Field Programmable Gate Array) implementation shows a great higher efficiency compared with ARM CPU and Pascal GPU, the detection speed is still slow and the memory is high when considering the small input resolution.

Feature descriptors like Histogram of Orientated Gradient (HOG), Local Binary Pattern (LBP), and Haar-like features are also proved to be efficient for vehicle detection [8,9]. As a consequence, those algorithms require much lower computational resources compared to CNN applications.

For object detection, a multi-scale detector always requires much more memory [10,11]. Usually, as the size of detection windows is fixed, to detect objects in different scales, a raw image needs to be buffered into different sizes and this process enhances the usage of storage (e.g., Static Random-Access Memory (SRAM)).

A sliding window is a common approach for object detection [12]. Classifiers will determine the similarity between the feature vector of the window and samples. A detection window is divided into some local regions (blocks and cells) to calculate feature vectors, while the window is shifting on the image, many cells and blocks are overlapped. One possible solution to avoid overlapping processing is to calculate each cell and use the results to construct the feature vector of blocks and construct a detection window. Meanwhile, feature-vector normalization among a block usually requires dividers. Traditional digital division methods cannot meet the speed requirement. A common way for the fast division is to create a look-up table to store the reciprocal of divisors and convert the division into a multiplication and table-lookup problem.

The hardware implementation of the HOG plus SVM (support vector machine) has been discussed to improve the speed-power performance [13]. Peng [14] studied the HOG feature extractor circuit for real-time detection and discussed how the sliding window size and cell size can affect the performance. To increase the robustness, the histograms of cells are combined and then normalized in L2 form to construct a descriptor of a block.

### 1.2. Contribution

**Figure 1:**
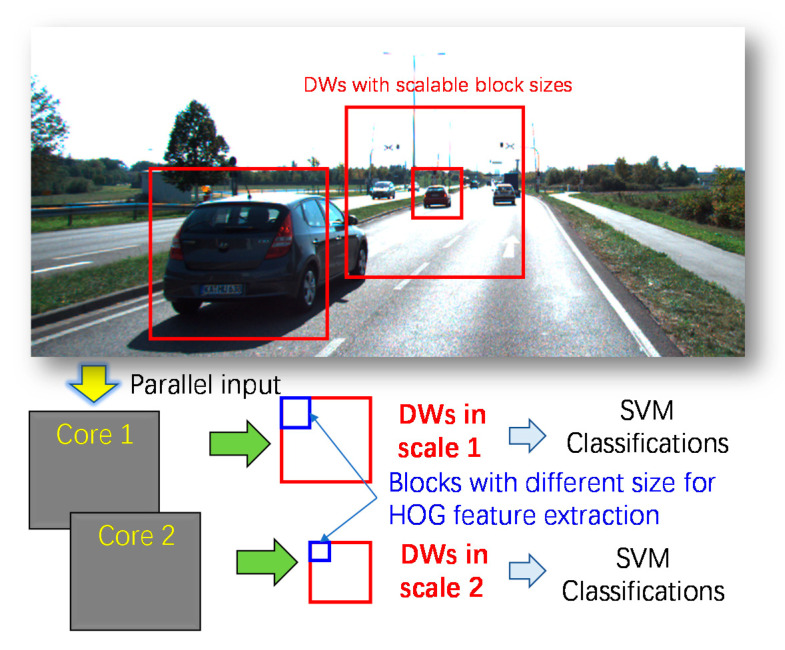
The overview of the proposed multi-core object detection coprocessor for multi-scale/type classification with the HOG and SVM framework.

In this paper, we propose a multi-core object detection coprocessor for multi-scale/type classification considering the speed-power-accuracy tradeoff within the HOG and SVM framework as shown in Figure 1. The contribution of this paper can be summarized into three aspects as follows: 

Firstly, differing from our previous work [15], a scalable size of a block is implemented in this work, which enables the flexibility to detect objects in different shapes and scales. For instance, a vertical rectangle detection window (DW) is often used in pedestrian detection but a horizontal rectangle DW is suitable for vehicle detection.

Then, a sliding detection window mechanism with a scalable block size enables parallel partial SVM classifications of all DWs that contain the being-processed block. This can avoid repeated computations of the overlapped blocks in different DWs and large memory for buffering DWs. Compared to the previous FPGA-implemented HOG-SVM classifiers [15,16,17,18] with only a dedicated scale, we provide multi-scale detection for vehicle detection in this work.

Finally, an approximation divider with the multiplication of the reciprocal using Taylor expansion solves the critical path of the normalization circuitry so that it can synchronize to the working frequency of the image sensor for low dynamic power consumption. In contrast to the general implementation of a divider, not only less hardware-resource usage but also the max working frequency of this approximation divider can be significantly improved from 28.66 MHz to 162.81 MHz with 0.682% accuracy loss.

### 1.3. Structure

The remaining of the paper is organized as follows. Section 2 introduces the framework with the HOG feature and SVM classifier. Section 3 presents the VLSI-oriented algorithm for the proposed framework. The experimental results are discussed in Section 3. Finally, we conclude the paper in Section 4.

## 2. VLSI-Oriented Hardware Algorithm for Feature Extraction

### 2.1. Block-Level Feature Extraction and Normalization Circuitry

The HOG feature extraction starts with dividing the input image into non-overlapping subcomponents, the so-called cells with the size of $w_{cell}$ and $h_{cell}$. The gradients are calculated for each pixel within each cell. The gradient orientation of all pixels in a cell is mapped to B bins for each cell. The histogram normalized in a block with C×C cells to increase robustness to the variations, e.g., texture and illumination, results in a final (B×C)-dimensional feature.

Suppose that the size of the detection window is $w_{\mathrm{DW}}$ and $h_{\mathrm{DW}}$, since the blocks are overlapped by a cell, each DW has m×n blocks where $m=(w_{\mathrm{DW}}/w_{\mathrm{cell}}-1)$ in horizontal and $n=(h_{\mathrm{DW}}/h_{\mathrm{cell}}-1)$ in the vertical. The final dimensionality of the feature vector (FV) of a DW is $\left(B\times C^{2}\right)\times m\times n$.

A linear SVM classifier in conjunction with the HOG feature is a popular solution for object detection except for deep neural networks [4]. The SVM classifier for a DW with a $\left(B\times C^{2}\right)\times m\times n$-dimensional FV is trained off-line by training samples with the same size as a DW, i.e., $w_{\mathrm{DW}}\times h_{\mathrm{DW}}$, so that the SVM weight has the same dimensionality as a DW, i.e., $\left(B\times C^{2}\right)\times m\times n$.

It can be observed that the dimensionality keeps the same when the size of cells, i.e., $w_{cell}$ and $h_{cell}$ increases or decreases in the same ratio to the size of the DW, i.e., $w_{\mathrm{DW}}$ and $h_{\mathrm{DW}}$. One main factor differencing from the previous works [15,16,17,18] is that the size of the DW in this work can be scaled. This is the reason that, rather than pyramid images, the designed HOG feature extraction module can produce different sizes of DWs with the same dimensionality and thus achieve the multi-scaled detection.

**Figure 2:**
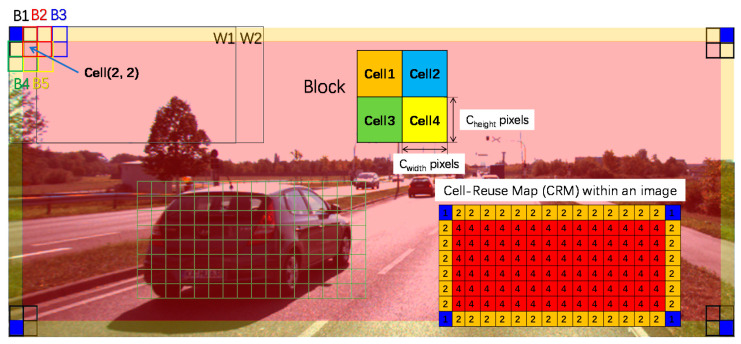
Illustration of the Cell-Reuse map based on the 128×64 DW and 2×2 cell blocks within an image.

For each block, a normalization step is applied to adjust the descriptors of cells in the block during the construction of the block’s local FV. Supposing that each block has 2 × 2 cells in Figure 2, block 1 (B1) and block 2 (B2) have two overlapped cells. It is observed that these two overlapped cells are reused two times in B1 and B2. Furthermore, the presence of, e.g., cell (2, 2) in four blocks (B1, B2, B4, and B5) leads to 4-fold reuse of this cell. Eventually, as shown in Figure 2, a cell-reuse map (CRM) within an image shows that corner cells, edge cells, and inter cells overlap one time, two times, and four times respectively.

Additionally, according to the CRM, the 2-D address of the cell in the image can derive the blocks which contain it and the locations (top-left, top-right, bottom-left, and bottom-right as shown in Figure 2) in the blocks. Then, the extracted cell-descriptor is added to the block descriptor memory for the block-level normalization. Since the blocks are overlapped by one cell, there are $w/w_{cell}-1$ blocks. Once the Cell (1, 1) has been extracted, the accumulation of Block 0 is then completed. At the same moment, the Cell (0, 0) stored in the cell line-buffer is read out for the normalization of Block 0. Accordingly, besides the first cell line, Cell (1, 0) and Cell (1, 1) must be stored in the cell line buffer. In the normalization module, the memory usage including the intermediate block memory and the cell line buffer is $w/w_{cell}\times {\mathrm{WL}}_{block}\times \;$ Bin + $\left(w/w_{cell}+2\right)\times {\mathrm{WL}}_{cell}\times \;$ Bin bits. Here, ${\mathrm{WL}}_{block}$ is the word length of the intermediate value of the cell accumulation for block normalization so that ${\mathrm{WL}}_{block}$ is equal to ${\mathrm{WL}}_{cell}+2$.

**Figure 3:**
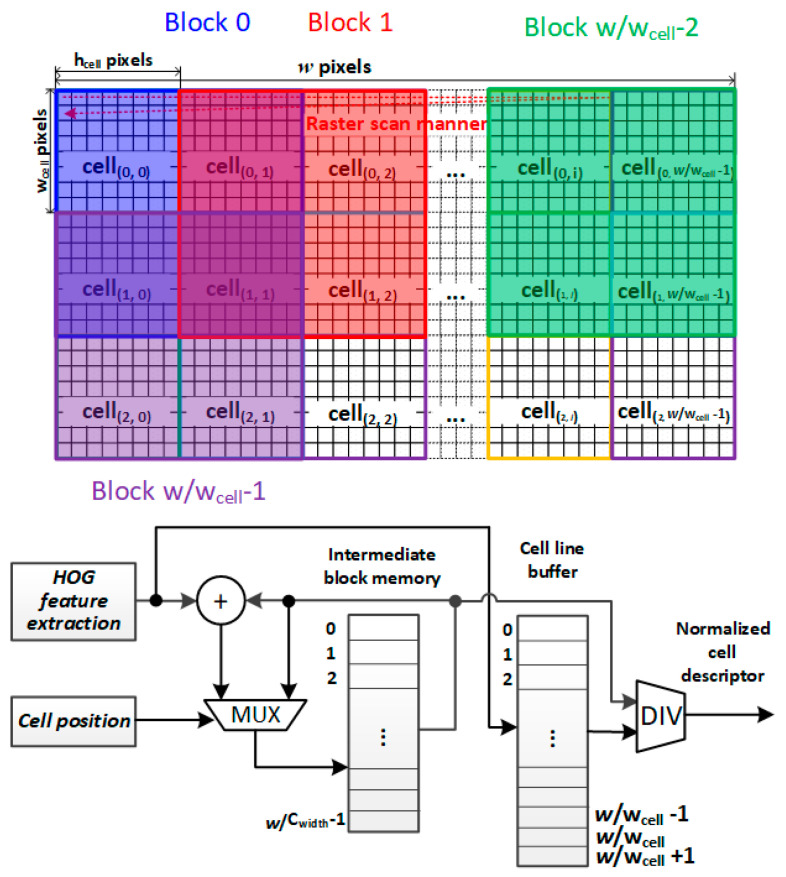
Block-level normalization. Cell position in a block and image is given by CRM.

In Figure 3, the HOG feature extraction unit has been implemented in our previous works [19] with the raster scan manner of the image sensor. It extracts the feature vectors of the cells from the image and the extracted cell vectors are then transferred into both the intermediate block memory and the cell line buffer. The cell position points to the location of the intermediate block memory to store the sum of the cells in their block. Once the sum of the cell vectors within a block is calculated, the feature vectors of the cells stored in the cell line buffer then divide this sum for normalization. Subsequentially, the normalized block feature is passed to the next module for partial SVM classification.

The critical path in the block normalization module is the divider. In this work, we consider the division as the multiplication of the dividend and the reciprocal of the divisor as Equation (1) where the reciprocal uses Taylor expansion. First, the divisor X is normalized into [1,2) as illustrated in Equation (2). Then we apply the first two series of Taylor expansion to estimate x as in Equations (3–5).

(1) $$\begin{array}{c} \frac{Y}{X}=Y\times \frac{1}{X} \end{array}$$

(2) $$\begin{array}{c} X=x\times 2^{j}\;\;\;\;\;\;\;\;x\in \left[1,2\right) \end{array}$$

(3) $$\begin{array}{c} f\left(x\right)=\frac{1}{x}\approx f_{i}\left(x_{i0}\right)+f_{i}^{\prime }\left(x_{i0}-x\right) \end{array}$$

(4) $$\begin{array}{c} f_{i}\left(x_{i0}\right)=\frac{1}{x_{i0}}\;\;\;f_{i}^{\prime }\left(x_{i0}\right)=\frac{1}{x_{i0}{}^{2}} \end{array}$$

To minimize the look-up-table of the $f_{i}\left(x_{i0}\right)$ and $\;f_{i}^{\prime }\left(x_{i0}\right)$, domain [1,2) is divided into n parts and we use the middle point $x_{i0}$ of each part to represent the value of the part in Equation (6). To make up for the loss of accuracy, Newton iteration [20] is used to increase the accuracy in a very efficient way. Finally, the division value can be given by Equation (7).

(5) $$\begin{array}{c} x_{i0}=1+i\times 2^{-n}-2^{-n-1} \end{array}$$

(6) $$\begin{array}{c} f_{2}\left(x\right)\approx f\left(x\right)\times \left(2-x\times f\left(x\right)\right) \end{array}$$

(7) $$\begin{array}{c} \frac{Y}{X}=Y\times \;f_{2}\left(x\right)\times 2^{-j} \end{array}$$

**Figure 4:**
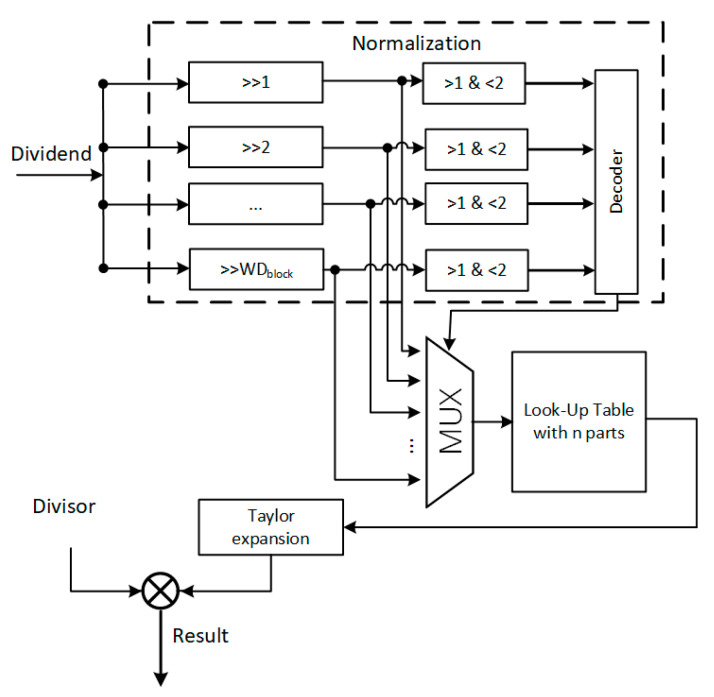
Optimized divider. The dividend is normalized into (1,2) and a lookup table is then used to estimate the Taylor expansion of the reciprocal of dividend.

As shown in Figure 4, the normalization unit is to map the dividend into a number within [1,2) after a bit-shifting. In particular, the look-up-table, in which the number within [1,2) is divided into n parts, determines the accuracy precision of the result. Of course, this is a tradeoff of the result accuracy and the hardware resource.

### 2.2. Scalable-Block-Size Based Sliding-Detection-Window (SBSSDW) Mechanism

Compared to our previous works in [19], in this work, the block-level normalization requires a larger memory but brings a smaller index look-up table and a lesser memory address calculation in the partial classification stage. For instance, a 128 × 64-pixel DW contains 420 cells but only 105 blocks so that the index and address number assignment of the overlapped DWs can be reduced to four times less than the cell-based method.

In particular, this work develops a scalable block size for multi-shape objects instead of the fixed cell/block size. For detecting objects in the input image, usually, the object will be detected by a sliding window with the overlapping of a block. Instead of the repeated computation of the overlapped cells and a large memory for buffering the window and the entire image, we develop a scalable-block-size-based sliding-detection-window (SBSSDW) mechanism in this work. For every pixel (m, n) and 0≤m<w,0≤n<h, it should be included in a block (i, j), where $⎣m/w_{block}⎦=i,\;⎣n/h_{block}⎦=j$. Consequently, we can model the index of the first window which contains the block (i, j) as shown in (10) to construct the window vectors. Here, each DW with the index ($i_{DW}$) is a 1-dimensional vector to represent the number of DWs overlapping by a block, i.e., $\left(w-w_{DW}/w_{block}+1\right)\times \left(h-h_{DW}/h_{block}+1\right)$. For example, a VGA image has 832 DWs while each DW has 128×64 pixels and each block contain 2 × 2 cells with 8 × 8 pixels. Hence, the index starts from 0 to 831.

(8) $$i_{FW}=\left|j-\frac{h_{DW}}{h_{block}}\right|\times \left(\frac{w-w_{DW}}{w_{block}}+1\right)+\left|i-\frac{w_{DW}}{w_{block}}\right|$$

(9) $$\begin{array}{c} \left(0\leq i\leq \frac{w-w_{\mathrm{DW}}}{w_{\mathrm{block}}},\;0\leq j\leq \frac{h-h_{\mathrm{DW}}}{h_{\mathrm{block}}}\right) \end{array}$$

Furthermore, the number of DWs that contains the block (i, j) can be calculated to decide the numbers of replications of this block, i.e., $\;N_{hor}\times \;N_{ver}$ where $\;N_{hor}$ in horizontal and $\;N_{ver}$ in vertical by Equations (10) and (11) respectively.

(10) $$\;N_{hor}=\{\begin{array}{c} i+1,\;\;0\leq \;i<⎡\frac{w_{DW}}{w_{block}}⎤\\ ⎡\frac{w_{DW}}{w_{block}}⎤,\;⎡\frac{w_{DW}}{w_{block}}⎤\leq i<⎡\frac{w}{w_{block}}⎤-⎡\frac{w_{DW}}{w_{block}}⎤\\ ⎡\frac{w_{DW}}{w_{block}}⎤-i,\;⎡\frac{w}{w_{block}}⎤-⎡\frac{w_{DW}}{w_{block}}⎤<i\geq ⎡\frac{w}{w_{block}}⎤ \end{array}$$

(11) $$\;N_{ver}=\{\begin{array}{c} j+1,\;\;0\leq \;j<⎡\frac{h_{DW}}{h_{block}}⎤\\ ⎡\frac{h_{DW}}{h_{block}}⎤,\;⎡\frac{h_{DW}}{h_{block}}⎤\leq j<⎡\frac{h}{h_{block}}⎤-⎡\frac{h_{DW}}{h_{block}}⎤\\ ⎡\frac{h_{DW}}{h_{block}}⎤-j,\;⎡\frac{h}{h_{block}}⎤-⎡\frac{h_{DW}}{h_{block}}⎤<j\geq ⎡\frac{h}{h_{block}}⎤ \end{array}$$

As Figure 2 shows, a block can be used in different DWs. After the block-level normalization, the FV of each block is used to construct the partial FV of the overlapped DWs. 

In the case of Block 3 in Figure 2, its index ($i_{FW}=$0) of the first window and the number of DWs ($\;N_{ver}\times \;N_{hor}=2\times 2=1$) containing Block (1, 1) can be calculated at the same moment based on the coordinate. The classification of the DWs is partially computed and the intermediate results are buffered in memory. This can significantly reduce memory usage and certainly reduce the delay in the classification of DWs. Once the FV of the last block of a DW0 is extracted, the classification for DW0 with a linear support vector machine (SVM) [8] can be completed with a short delay. 

The linear SVM approach aims to construct a classifier which can be mathematically represented like $y\left(\vec{v}\right)=\mathrm{sgn}\left[f\left(\vec{v},\vec{w}\right)\right]$. Here, $\vec{v}$ and $\vec{w}$ represent the FV of DWs and weight vector, $f\left(\vec{v},\vec{w}\right)$ is the kernel function and sgn(·) is the sign function. The FVs of DWs were mapped into two classes, positive and negative by the classifier $y\left(\vec{v}\right)$ and then the cost function would be used to evaluate the cost between the label of the $\vec{v}$ and $y\left(\vec{v}\right)$.

The SVM prediction part uses the parameters from the off-line trained model to classify the class of a DW. The weight vector $\vec{w}$ and the bias b are the two essential components from the trained linear SVM model, which is initially stored in the dual-port memory (DPM) in advance. Then the FV of a DW multiply-accumulated the weight vector and finally adds the bias b to predict the positive or negative class. 

**Figure 5:**
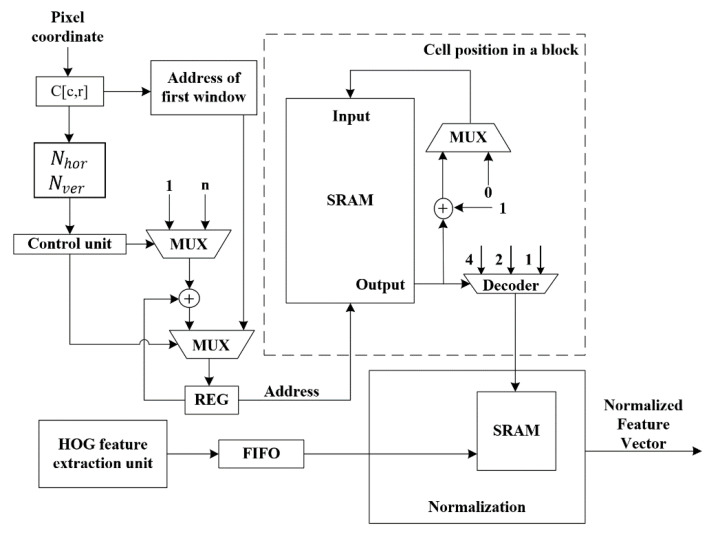
Hardware architecture of SBSSDW, the pixel index, $\;N_{hor}$ and $N_{ver}$ is calculated by this module.

In the hardware architecture (Figure 5) for SBSSDW, the pixel coordinates are converted to the position of the processed cells and blocks in a frame for calculating the corresponding index of the first window, $\;N_{hor}$ and $N_{ver}$. In the beginning, the index of the first window is set to $i_{FW}$ and the index then increases after handling the components of block-level FVs from $i_{FW}$ to $i_{FW}+\;N_{hor}$ horizontally. When the processing reached the end of a row, the index is reassigned to $i_{FW}+x\times n$ where $n=\left(w-w_{DW}\right)/W_{block}$ representing the maximum overlapped sliding window in the horizontal direction of a frame and x is the row number of the DW. Finally, the classification of a block ends until the index reaches $i_{FW}+\;N_{hor}+\left(N_{ver}-1\right)\times n$.

For a block, its corresponding components of FVs of a DW are readout. Meanwhile, the classification intermediate values of the DW stored in the SRAM are firstly read out to continually compute the SVM classification. The classification will be completed if this block is the final pitch of the DW. The above operation is then repeated until all DWs contain this block. As a consequence, the block should be stored in a FIFO.

It can be observed that the classification processing starts at the last pixel-row of a frame and need to be completed in $w\times h_{block}$ clocks. The feature-extraction and block-level normalization unit are synchronized with the working frequency of the image sensor. Whereas, a buffer for block FVs, i.e., FIFO, has to be used to store the unprocessed data. Hence, this causes a tradeoff between the buffer size and the power dissipation. The buffer size can be significantly reduced when the SVM classification module adopts a higher working clock frequency but with high power dissipation. On the other hand, the working frequency can synchronize to the HOG feature and normalization module when the classification DWs for a block under processing is parallelized. In the case of the maximum parallelism with $⎡h_{DW}/h_{block}⎤\times ⎡w_{DW}/w_{block}⎤$, although the FIFO does not need to be set, the memory of the weight for SVM requires $⎡h_{DW}/h_{block}⎤\times ⎡w_{DW}/w_{block}⎤$ ports. As a consequence, parallelism defines the complexity of the hardware resource in the SVM module.

### 2.3. Multi-Scale Detection with Multi-Core Implementation

Usually, an image pyramid is generated for each frame for supporting multi-scale detection. In the case of a single core, besides the large buffer for scaled images, a high working frequency must be applied to process these additional scales.

In this work, a multi-core architecture can avoid the frame buffer memory and a high working frequency. Each core detects objects with a scaled DW by adjusting the width and height of the cell in the HOG feature extraction module as described in Section 3.1 and Figure 2. For each core, the key parameters vary because of the different sizes of sliding windows. In other words, each core will construct their map of reuse times of each cell, the index of each cell, and the feature vectors. The number of cores is equivalent to the number of scaled images to detect objects in different sizes while each core has the same on the hardware structure.

## 3. Experiment Results and Discussion

### 3.1. Hardware Implementation and Performance Analysis

**Figure 6:**
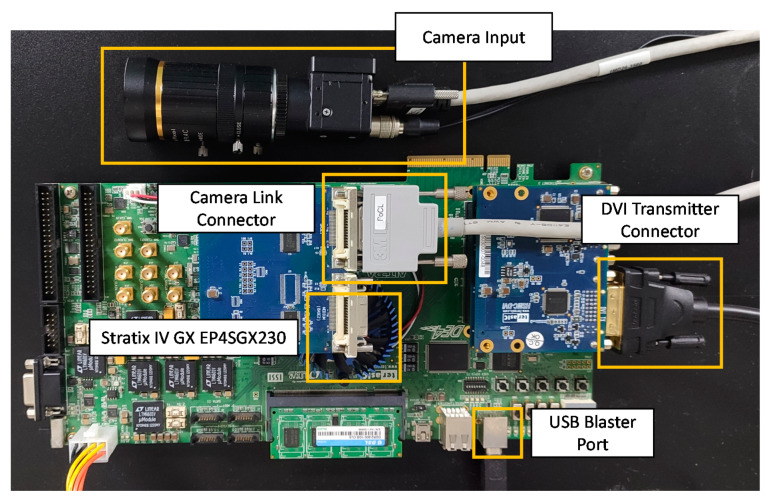
FPGA-based prototype system deployed on the Intel DE4 development board (Stratix IV GX EP4SGX230).

To estimate the performance, we deployed this work on the Intel DE4 development board (Stratix IV GX EP4SGX230 [21]) with an STC-MC83PCL [22] camera-input XGA signal (1024 × 768 @ 60 Hz), as shown in Figure 6. In this work, the image size is constrained by two factors: w the width of the line buffer in the Sobel filter module and $DP_{cellmem}$, the depth of the memory for storing the intermediate FVs of cells in a row in Figure 2, i.e., $DP_{cellmem}=w/w_{cell}$. As illustrated in Figure 5, synchronized with the pixel-based HOG feature extraction, the pixel coordinates are also converted to the position of the processed cell in the image frame for the simultaneous calculation of the corresponding index of the first window $i_{FW}$, $N_{hor}$ and $N_{ver}$. The weight of the offline trained SVM classifier is initialized in the DPM.

### 3.2. Discussion and Comparison

We define the width of the line buffer w = 2048 and the depth of the cell FV memory $DP_{cellmem}=256$. Table 1 list the resources, working frequency, and power dissipation for different modules. Most of the intense computing process is the normalization and SVM module. To avoid data overflow between modules and large FIFO due to different processing speeds, the working frequency of the SVM module must be two times faster than the Sobel filter, HOG feature, and the normalization module. The working frequency of Sobel, HOG, and normalization can be synchronized with the pixel clock, which is 65 MHz for XGA. The max working frequency of the Sobel filter and the HOG feature can easily satisfy this speed requirement. In particular, the maximum working frequency of the Quartus LPM divider IP core, e.g., the 18-bit divider with 28.66 MHz, is hard to meet the speed for real-time processing. In this work, we thus improve the critical path caused by the divider in the normalization module using Taylor expansion. The accuracy loss of the divider is only 0.682% and while this is small enough, it has a limited effect on the SVM prediction. Meanwhile, the max working frequency of the normalization module has been improved to 162.81 MHz.

The power consumption of each module and the whole design estimations are shown in Table 1 where it is estimated using Quartus Power Estimator 18.1 with their max working frequency. In the case of the same working frequency, the powers of the Sobel Filter, HOG Feature, and normalization parts increase gradually because of the increased resource usage of each module. This can explain why the SVM part consumes the highest power, i.e., its highest clock frequency and most resource usage.

By using the SBSSDW, only a small part of the intermediate FVs need to be stored for SVM prediction and finally, we reduced the total SRAM usage to 281 Kbit, in which 73.7 Kbit is used to store the weight for SVM classification. Consider that the max working frequency of SVM, 136.43 MHz, and the detection speed is 60 fps for an XGA (1024 × 768 pixels) video input or 30 fps for a 2K (2048 × 2048) video. Compared with other related work as shown in Table 2, our work uses significantly less memory than [16]. Additionally, the hardware resource with the throughput of 60 fps XGA video of this work is only one-tenth that of [17] with a throughput of 60 fps VGA.

**Figure 7:**
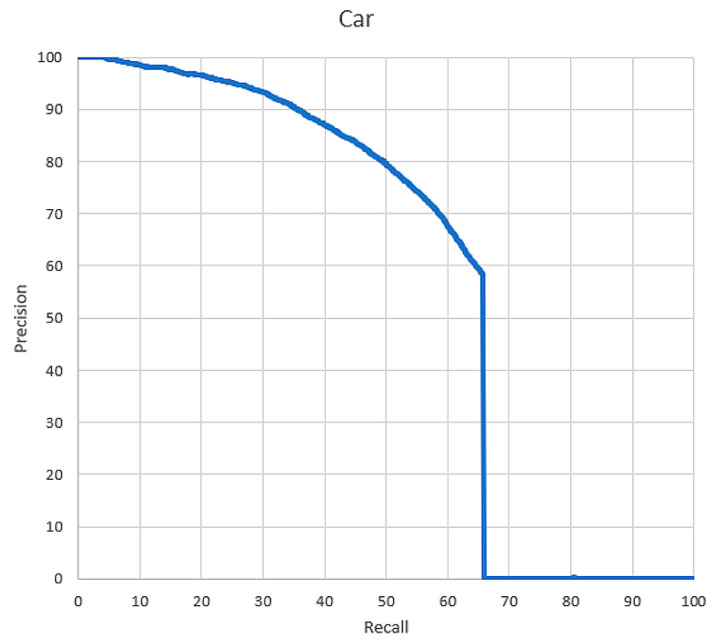
The precision-recall curve of our vehicle detection classifier. For a required recall, if our result can reach the recall, the corresponding precision can be calculated according to the measurement criteria in [23].

We are using the KITTI object detection suite [23] as our database, which contains 7481 training images and 7518 test images, comprising a total of 39,595 labeled objects (including cars, pedestrians, and cyclists). As we focused on detecting cars, we picked out 6996 samples from all the 33,259 labeled cars as our positive training samples and randomly extracted 40,000 carless samples from the large picture as our negative training samples. Classification results are simulated by the software implementation and the results are illustrated in Figure 7. To quantify the performance of our implementation, we plot the precision-recall curve (Figure 7) with the measurement criteria discussed in [23], which is the formal evaluation mechanism of the KITTI dataset and the average precision of our result is 52.07%, where the error rate indicates that the detected bounding boxes are correct only if they overlap by at least 50% with a ground truth bounding box. This average precision ranks at 316th in the car precision list of KITTI except for the speed performance due to the hardware implementation. 

**Figure 8:**
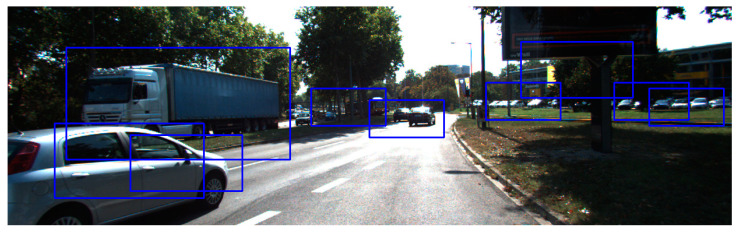
Detection example in the scenario with complex edge information of buildings and background-appearing vehicle-like outlines.

This result implies that it is difficult to detect vehicles in a relatively complex environment because of the inherent property of the HOG feature, which is sensitive to the complex edge characteristic information. As shown in Figure 8, the classifier not only detects the real vehicles but also treats some buildings and backgrounds as the vehicle. The complex environment means that the scenario with edge information of buildings and background appear as vehicle-like outlines. Further work needs to be conducted to detect vehicles in a complex environment with complex feature combinations.

Since the size of the DW is scalable, in this work, we compare the classification result of both the 64 × 128 (1:2) and the 128 × 64 (2:1) detection windows. Normally, the SVM classifier is trained by a 1:2 scaled window for pedestrian detection, but in our design, we found that the shape of the detection window can significantly influence the result of classification since the orientation of cars is different compared with pedestrians. Finally, we chose to use a 2:1 scaled window with different sizes to detect car objects.

## 4. Conclusions

This paper presents a hardware-friendly architecture on FPGA that implements a cell-based Histogram of Oriented Gradient (HOG) feature extraction circuitry, a block-level normalization unit, and a partial Support Vector Machine (SVM) classifier module within a scalable-block-size-based sliding-detection-window (SBSSDW) mechanism. Within the SBSSDW method, the feature vector of each detection window (DW) can be constructed according to the location of the block in a window without a large buffer. Additionally, the proposed architecture is capable of the scalable size of the DW from 32 × 32 up to 2048 × 2048 pixels. The experimental results show that the coprocessor attains an average precision of 52.07% with the power dissipation of 80.98 mW. The accuracy performance implies that the complex edge characteristic information significantly affects the HOG feature. Thus, a feature combination is to be conducted to detect objects in a complex environment in our further work.

## Figures and Tables

**Table 1:** Hardware resources of each module *.

	Sobel Filter	HOG Feature	Normalization	SVM	Total
Combinational ALUTs	442	549	4678	3017	8686
Dedicated Logic Registers	616	587	1694	5319	8216
Block Memory Bits	18,432	49,152	39,616	174,080	281,280
Max Working Frequency	356.63 MHz	323.31 MHz	162.81 MHz	136.43 MHz	
Actual Working Frequency	65 MHz	65 MHz	65 MHz	130 MHz	
Power Consumption	2.84 mW	3.51 mW	9.47 mW	65.17 mW	80.98 mW

* All the resources are measured by Quartus Premium 18.1.

**Table 2:** Resource comparison.

	[16]	[17]	[18]	[15]	This Study
Combinational ALUTs	85,837	87,306	6551	7652	8686
Dedicated Logic Registers	406,978	77,726	4375	4503	8216
Block Memory Bits (Mbit)	2.55	2.97	NA	0.13	0.28
Max Working Frequency (MHz)	135	108.19	50	* NA	130
Frame rate (fps)	68.18	60	25	60	60
Resolution	640 × 480	640 × 480	640 × 480	1920 × 1080	1024 × 768
FPGA platform	Virtex-6	Cyclone IV		Stratix IV	

* NA means this value is not listed in the reference paper.
